# Combined effects of thermal stress and benzene or toluene exposure on the immune and reproductive disruption in Nile tilapia (*Oreochromis niloticus*)

**DOI:** 10.1038/s41598-026-66156-2

**Published:** 2026-08-19

**Authors:** Hossam A. AboElkhair, El-Sabry Abu Amra, Fathy M. Elshaer, Hamdy A. M. Soliman, Alaa El-Din H. Sayed

**Affiliations:** 1 Department of Zoology, Faculty of Science, Sohag University, Sohag, 82524 Egypt; 2 Department of Zoology, Faculty of Science (girls), Al-Azhar University, Assiut, 71516 Egypt; 3 Department of Zoology, Faculty of Science, Assiut University, Assiut, 71516 Egypt; 4 Molecular Biology Research & Studies Institute, Assiut University, Assiut, 71516 Egypt

**Keywords:** Nile tilapia, Thermal stress, Benzene, Toluene, Immune response, Endocrine disruption, Fibrosis, Ecology, Ecology, Environmental sciences, Immunology, Physiology, Zoology

## Abstract

Thermal stress and exposure to benzene or toluene frequently co-occur in aquatic environments; however, information on their combined effects on fish physiology remains limited. Therefore, this study examines the interactive impacts of high temperature and benzene or toluene exposure on the immune and reproductive systems of Nile tilapia (*Oreochromis niloticus*). Fish were assigned to six experimental groups: the first group served as the control (29 ± 1 °C), the second group was exposed to thermal stress (34 ± 1 °C), the third and fourth groups were exposed to benzene (6.1 mg/L) and toluene (5.9 mg/L), respectively, at 29 ± 1 °C, and the fifth and sixth groups were exposed to benzene (6.1 mg/L) and toluene (5.9 mg/L), respectively, at 34 ± 1 °C. All treatments were conducted over a two-week period. The findings showed that combined exposure caused greater immunological and reproductive alterations than individual stressors, with more pronounced decreases in lysozyme (LYZ), immunoglobulin M (IgM), follicle-stimulating hormone (FSH), luteinizing hormone (LH), and testosterone (T) levels, and greater increases in interleukin-1 beta (IL-1β) and interleukin-6 (IL-6) concentrations. Histopathological examination revealed severe splenic lesions, including necrosis, vascular changes, and hemosiderin deposition, together with severe testicular damage characterized by seminiferous tubule degeneration and complete atrophy of sperm cells, accompanied by greater fibrosis in both organs compared with the individual stressor groups. These findings indicate that the combined effects of elevated water temperature and benzene or toluene contamination may compromise immune competence and reproductive capacity in fish, highlighting potential risks to aquatic populations under ongoing environmental change.

## Introduction

The accelerated pace of economic growth has increased the release of multiple environmental stressors into aquatic ecosystems^1^. Among these stressors, chemical contamination and rising water temperatures associated with climate change represent major threats to aquatic organisms^2^. Aromatic hydrocarbons, particularly benzene and toluene, are frequently detected aquatic contaminants due to their extensive industrial applications, environmental mobility, and potential ecological toxicity^3,4^. Once accumulated in aquatic organisms, these compounds can disrupt multiple physiological systems, including neurological, cardiovascular, hepatic, renal, hematological, reproductive, and immune functions^5–11^.

Benzene has received considerable attention because of its immunotoxic and reproductive effects^12,13^. It suppresses immune cell populations and damages the spleen, leading to impaired host defense^14,15^. It also impairs splenic architecture, leading to increased transgene mutations, reduced lymphocyte counts, and weakened host defense against pathogens^16,17^. Benzene also disrupts spermatogenesis and endocrine signaling, thereby compromising reproductive performance^18–24^. Likewise, toluene disrupts immune homeostasis by modulating lymphocyte-mediated immune responses^25,26^ and impairs reproductive function through testicular degeneration and disruption of steroidogenesis^27,28^. These reproductive effects are partly mediated through suppression of T synthesis via downregulation of 3β-hydroxysteroid dehydrogenase expression^29–32^.

Climate-driven warming may further amplify the biological effects of chemical pollution, emphasizing the importance of evaluating multiple environmental stressors simultaneously^33^. Thermal stress disrupts immune homeostasis and promotes splenic inflammation^34,35^. It also impairs reproductive function by disrupting gamete maturation, spermatogenesis, and sex hormone regulation, ultimately reducing reproductive success^36–40^.

Changes in immune and reproductive functions, together with histopathological alterations, are widely recognized as sensitive biomarkers for assessing the physiological impacts of environmental stressors in aquatic organisms^12,41–43^.

Nile tilapia is one of the world’s most widely cultured fish owing to its economic importance, favorable biological traits, and sensitivity to environmental contaminants, making it a valuable model for toxicological studies^44,45^.

Understanding how multiple environmental stressors interact is essential for predicting the ecological consequences of environmental change and supporting sustainable aquatic ecosystem management^46,47^. Climate change is expected to alter the environmental fate, exposure, and toxicity of chemical contaminants, increasing the simultaneous exposure of aquatic organisms to elevated temperatures and pollutants^48–50^. Consequently, aquatic organisms are increasingly exposed to elevated temperatures together with benzene or toluene, raising concerns that thermal stress may modify their biological effects. Although the individual effects of thermal stress, benzene, and toluene have been extensively investigated, their combined effects on immune and reproductive functions and histopathological alterations in fish remain poorly understood. To address this knowledge gap, the present study investigated the combined effects of elevated water temperature and benzene or toluene on immune and reproductive functions and histopathological alterations in Nile tilapia. We hypothesized that combined exposure to thermal stress and benzene or toluene would result in enhanced physiological and histopathological alterations compared with exposure to either stressor alone.

## Materials and methods

### Chemicals

Analytical-grade benzene (CAS No. 71-43-2) and toluene (CAS No. 108-88-3), both with 99.8% purity, were purchased from Sigma-Aldrich (Cairo, Egypt).

### Animal ethics statement

All experimental procedures involving Nile tilapia were conducted in accordance with the guidelines for the care and use of laboratory animals and were approved by the Institutional Ethics Committee of Sohag University, Egypt (Approval No. CSRE-22–25).

### Fish

Healthy male Nile tilapia (body weight 27 ± 8 g and total length 10.7 ± 0.9 cm) were obtained from the aquaponics system of the Fish Biology and Pollution Laboratory, Faculty of Science, Assiut University, Egypt. Fish were sexed before the beginning of the experiment, and only males were selected based on external morphological examination to minimize variation associated with sex differences in reproductive parameters. Fish were examined for external and internal parasitic infections by visual inspection of external surfaces (skin, fins, and gills) and skin mucus smears according to the guidelines of the American Fisheries Society, Fish Health Section^51^. After transferring to the laboratory, fish were acclimated for 30 days in aerated glass aquaria (100 × 70 × 50 cm) containing dechlorinated tap water. During the acclimation period, fish were maintained in 18 identical aquaria at a stocking density of 10 fish per aquarium. Fish were fed a commercial floating pelleted diet containing 30% crude protein (ALLER TIL-PRO 30% CP; Aller Aqua Egypt) twice daily at 5% of their body weight throughout the acclimation and experimental period. Half of the water volume was renewed daily. During the acclimation period, fish were maintained at 29 ± 1 °C under a 12 h:12 h light–dark photoperiod. Water quality parameters were monitored regularly throughout the acclimation and experimental periods. Dissolved oxygen was maintained at 6.9 mg/L, pH was 7.4, conductivity was 260.8 µS/cm, ammonia was 0.014 ± 0.001 mg/L, and nitrate was 0.178 ± 0.041 mg/L.

### Experimental groups

The fish were randomly distributed into six experimental groups, each consisting of 30 fish. For each treatment, fish were equally allocated among three replicate aquaria (*n* = 3; 10 fish per aquarium). All replicate aquaria were identical in size and maintained under the same environmental conditions. The aquarium was considered the experimental unit for statistical analyses, with two fish were randomly collected from each replicate aquarium, resulting in a total of six fish per treatment for subsequent analyses.

**G1: Control**: maintained at 29 ± 1 °C.

**G2: Thermal stress**: exposed to high temperature (34 ± 1 °C).

**G3: Benzene only**: maintained at 29 ± 1 °C with benzene (6.1 mg L⁻¹).

**G4: Toluene only**: maintained at 29 ± 1 °C with toluene (5.9 mg L⁻¹).

**G5: Thermal stress + benzene**: exposed to 34 ± 1 °C and benzene (6.1 mg L⁻¹).

**G6: Thermal stress + toluene**: exposed to 34 ± 1 °C and toluene (5.9 mg L⁻¹).

All experimental groups were exposed to their respective treatments for 14 consecutive days. This exposure period was selected because it falls within the subacute exposure duration^52^ and has been previously applied in studies investigating thermal stress under comparable experimental conditions^53^.

### Exposure protocol

Water temperature was maintained using thermostatically controlled heaters and continuously monitored with calibrated digital thermometers. Temperature measurements were recorded during both daytime and nighttime to ensure stability throughout the experimental period^54^. For the thermal stress groups, the water temperature gradually increased from 29 ± 1 °C to 34 ± 1 °C at a rate of approximately 1 °C per hour to minimize thermal shock. Benzene and toluene exposure concentrations were prepared from analytical-grade stock solutions and added directly to the experimental water at the desired nominal concentrations without the use of a carrier solvent. Exposure concentrations were renewed daily by replacing half of the water volume with fresh filtered, dechlorinated water and adding an amount of benzene or toluene equivalent to half of the nominal exposure concentration to the replacement water, according to Sayed et al.^3^. This daily renewal procedure was used to compensate for any potential loss of benzene and toluene due to volatilization and to maintain the nominal exposure concentrations throughout the experimental period. The exposure concentrations of benzene (6.1 mg/L) and toluene (5.9 mg/L) were selected according to a previous toxicological study^55^. The temperature of 34 ± 1 °C was selected based on previous studies conducted under comparable experimental conditions in Nile tilapia, where this temperature was used as acute thermal challenge to induce physiological stress^53,56^.

### Sample collection

At the end of the 14-day exposure period, six fish were randomly collected from each treatment for subsequent analyses, with two fish sampled from each of the three replicate aquaria. and anesthetized in ice-cold water to reduce handling stress, as described by Hamed et al.^57^. Fish were subsequently euthanized by prolonged immersion in ice-cold water until loss of consciousness and cessation of opercular movements, following approved ethical guidelines. The blood was taken out of the caudal vein then left to coagulate. Centrifugation was performed at 5000 rpm in 20 min, and serum was utilized in the analysis of reproductive and immunological parameters. All serum samples were analyzed in triplicate to ensure the accuracy and reliability of the obtained results. Following the blood collection, spleen and testis tissues were aseptically removed of each fish and stored to undergo histopathological analysis.

### Immunological parameters

Serum samples were stored at − 80 °C until analysis of immunological parameters. All ELISA kits used for immunological analyses were obtained from Diagnostics Biochem Canada Inc., London, Ontario, Canada. LYZ was determined utilizing a turbidity assay following Parry Jr et al.^58^ and the procedure described by Overkamp et al.^59^. IgM was quantified through an ELISA kit (CSB-E12045 F h) according to the manufacturer’s instructions. IL-1β and IL-6concentrations were determined utilizing ELISA kits (CSB-E13259 F h and CSB-E13258 F h) according to the manufacturer’s instructions and the methods of Wang et al.^60^ and Hanington and Belosevic^61^, respectively.

### Reproductive parameters

Reproductive hormones were quantified by ELISA kits (Diagnostics Biochem Canada Inc., London, Ontario, Canada). T was determined utilizing kits (Cat. Nos. CANE-1430 and CAN-TE-250) following Check et al.^62^,. FSH and LH were determined using kits (Cat. Nos. CANFSH-4060 and CAN-LH-4040) as reported by Knobil^63 ^and Cumming et al.^64^, respectively.

### Histopathological analysis

For each fish, three histological sections were prepared and examined from each tissue for histopathological evaluation. Each fish’s spleen and testis were removed and cleaned with physiological saline solution before getting fixed in neutralized buffered formalin. Following dehydration using an ascending series of ethanol concentration, treated with methyl benzoate for clearing, and then in paraffin embedding after which 7 μm thick sections were subsequently obtained, dewaxed, rehydrated, followed by staining with hematoxylin and eosin (H&E) enabling general histological evaluation following Feldman and Wolfe^65^. Addition sections stained with Sirius Red to visualize collagen fibers^66^. H&E stained sections were evaluated qualitatively based on the observed histopathological alterations in each experimental group. In contrast, Sirius Red-stained sections were subjected to quantitative image analysis to measure collagen deposition. For each fish, three histological sections from each tissue were examined for histopathological evaluation. Histological and histochemical analyses were performed using a light microscope (Olympus CX31, Japan) supplied with a digital camera (Toup Tek Toup View, Version ×86, China). Digital images of Sirius Red-stained sections were captured under the same magnification and illumination conditions and analyzed using ImageJ software. Quantitative analysis of collagen deposition was performed by measuring the collagen-positive area (%) in randomly selected non-overlapping fields using the color threshold function of ImageJ. The mean value of the analyzed fields was calculated and used for statistical analysis.

### Statistical analysis

Data were reported as mean and standard error (SE). The statistical analyses were done using IBM SPSS Statistics (version 25). The aquarium was considered the experimental unit for all statistical analyses. Three replicate aquaria were used for each treatment, and two fish were randomly sampled from each aquarium for the subsequent analyses. The Levene test was applied to assess variance homogeneity, whereas the Shapiro Wilk test was utilized to test data normality. The group means were compared using one-way analysis of variance (ANOVA), followed by Tukey’s post hoc test. The values that had different superscript letters (a, b, c,) were regarded as significantly different (*P* < 0.05). GraphPad Prism software (version 8.0.0; GraphPad Software, San Diego, CA, USA) was utilized to create graphs www.graphpad.com.

## Results

### Immunological parameters

**Fig. 1:**
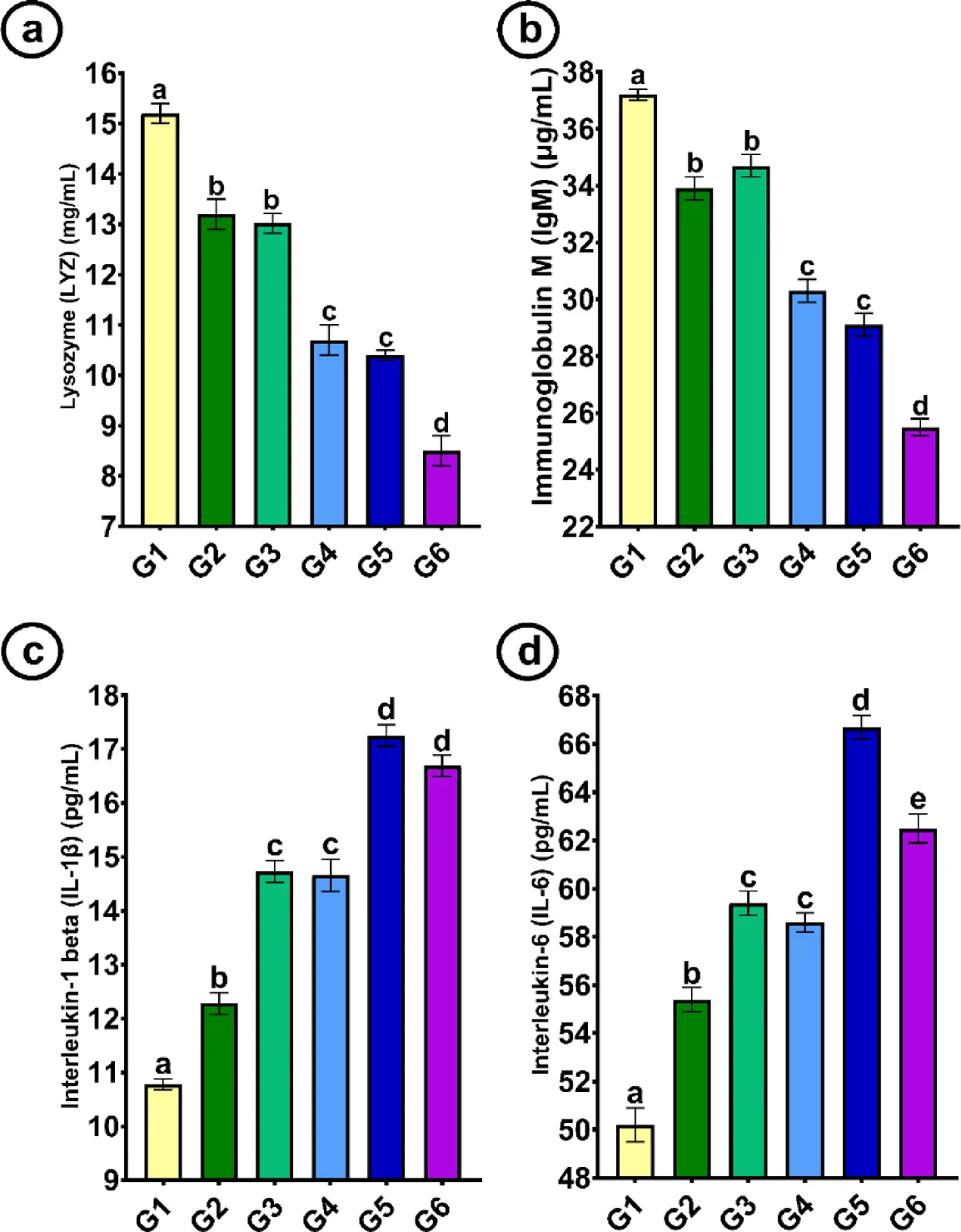
The effect of elevated temperature on the levels of LYZ, IgM, IL-1β and IL-6 in *O. niloticus* under aromatic hydrocarbons exposure. G1: Control (29 ± 1 °C); G2: Thermal stress (34 ± 1 °C); G3: Benzene (29 ± 1 °C + 6.1 mg L⁻¹); G4: Toluene (29 ± 1 °C + 5.9 mg L⁻¹); G5: Thermal stress + benzene (34 ± 1 °C + 6.1 mg L⁻¹); and G6: Thermal stress + toluene (34 ± 1 °C + 5.9 mg L⁻¹). Data are presented as mean ± SE (*n* = 6 fish per group). Statistical differences among groups were analyzed using one-way ANOVA followed by Tukey’s post hoc test. Bars with different letters indicate significant differences among groups at *P* < 0.05.

The immunological parameters of *O. niloticus* under different experimental conditions are presented in Fig. 1. LYZ and IgM levels were significantly lower in all single-stressor groups (thermal stress, benzene, and toluene) compared with the control group (*P* < 0.05). No significant difference was observed between the thermal stress and benzene groups (*P* > 0.05), whereas the toluene group showed significantly lower LYZ and IgM levels compared with the other single-stressor groups (*P* < 0.05). The combined exposure groups (thermal stress + benzene and thermal stress + toluene) exhibited significantly lower LYZ and IgM levels compared with the control and respective single-stressor groups (*P* < 0.05). Significant differences were also detected between the two combined-stressor groups for LYZ and IgM levels (*P* < 0.05) (Fig. 1a, b).

The IL-1β levels were significantly higher in all treated groups compared with the control group (*P* < 0.05). Among the single-stressor groups, the thermal stress group showed significantly lower IL-1β levels compared with the benzene and toluene groups (*P* < 0.05), while no significant difference was observed between the benzene and toluene groups (*P* > 0.05). The combined exposure groups (thermal stress + benzene and thermal stress + toluene) showed the highest IL-1β levels, which were significantly higher than those of the control and respective single-stressor groups (*P* < 0.05). No significant difference was observed between the two combined-stressor groups (*P* > 0.05) (Fig. 1c).

The IL-6 levels were significantly higher in all treated groups compared with the control group (*P* < 0.05). The thermal stress group showed significantly lower IL-6 levels compared with the benzene and toluene groups (*P* < 0.05), while no significant difference was detected between the benzene and toluene groups (*P* > 0.05). The combined exposure groups exhibited the highest IL-6 levels, which were significantly higher than those of the single-stressor and control groups (*P* < 0.05). A significant difference was observed between the two combined-stressor groups (*P* < 0.05) (Fig. 1d).

### Histopathological and histochemical studies of spleen

**Fig. 2:**
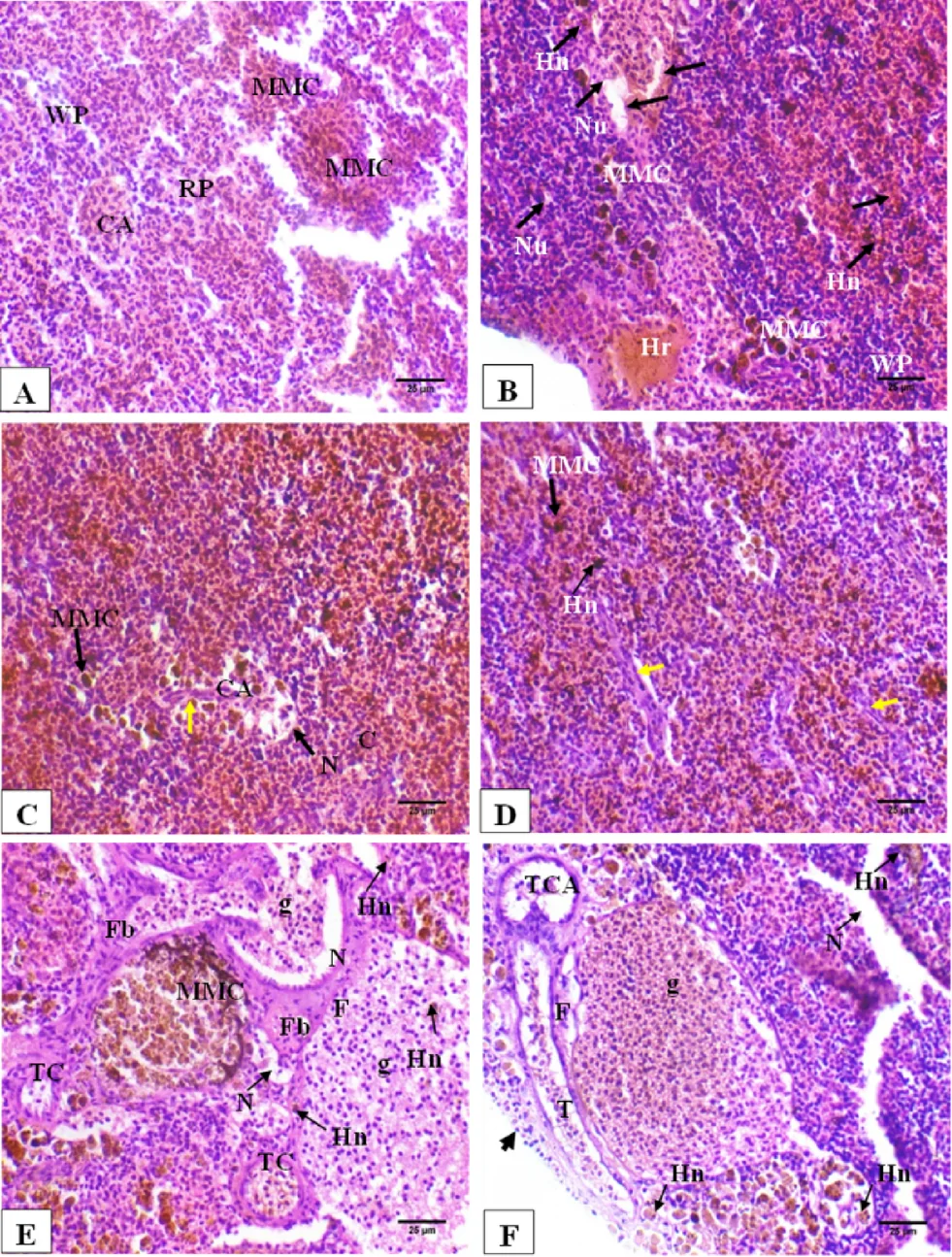
**(A)** Light micrographs of *O. niloticus spleen* sections showing normal histological architecture of control. white pulp (WP), Red pulp (RP), Melano macrophage center (MMC), central arteriole (CA), were observed. **(B)** Exposure to thermal conditions showing hemorrhage (Hr) and hemosiderin (Hn) in splenic tissue, Black arrow denotes excess sinusoidal spaces, neutrophilic infiltration (Nu), Melano macrophage center (MMC), white pulp (WP), central arteriole (CA). **(C)** Spleen sections exposed to benzene showing elongated nucleus of smooth muscle cell lining blood vessel wall (yellow arrow), Melano macrophage center (MMC), central arteriole (CA) and Necrosis (N). (D) Spleen sections exposed to toluene showing elongated nucleus of smooth muscle cell lining blood vessel wall (yellow arrow), hemosiderin (Hn) in spleenic tissue; Melano macrophage center (MMC). **(E)** Spleen sections exposed to thermal stress & benzene showing granuloma (g) and the whole granuloma encapsulated with delicate layer of fibrous connective tissue (F), thickened clogged central arteriole (TCA), Melano macrophage center (MMC), Necrosis (N), fibrosis (Fb) and hemosiderin (Hn) in spleenic tissue was observed. **(F)** Spleen sections exposed to thermal stress & toluene showing Capsule (thick black arrow), trabeculae (T), granuloma (g) encapsulated with delicate layer of fibrous connective tissue (F), thickened clogged central arteriole (TCA), Necrosis (N), fibrosis (Fb) and hemosiderin (Hn) in spleenic tissue was observed. **H&E.**,** scale bar: 25 μm.**

Light micrographs of *O. niloticus* spleen sections showed the normal histological structure of the control, with distinct white pulp, red pulp, Melano macrophage centers, and a clear central arteriole (Fig. 2A). In comparison to the control group, spleen sections of fish exposed to elevated temperature had severe pathological changes, such as hemorrhage and hemosiderin deposition in the splenic tissue. Sinusoidal spaces, neutrophilic infiltration, Melano macrophage centers, white pulp, and central arteriole were also increased (Fig. 2B). Fish exposed to benzene alone had intermediate histopathological changes, such as elongation of smooth muscle cell nuclei lining blood vessel walls, Melano macrophage centers, central arterioles, and focal necrosis (Fig. 2C). Similarly, sections of the spleen of fish exposed to toluene alone exhibited elongation of smooth muscle cell nuclei lining the blood vessel walls, as well as hemosiderin deposition and Melano macrophage centers in the splenic tissue (Fig. 2D). In contrast, combined thermal stress and benzene exposure resulted in severe splenic damage in fish, which was characterized by the presence of granulomas surrounded by a delicate layer of fibrous connective tissue, thickened and clogged central arterioles, extensive necrosis, fibrosis, and hemosiderin accumulation in the splenic tissue (Fig. 2E). Likewise, sections of the spleen of fish subjected to combined thermal stress and toluene showed significant pathological alterations, such as capsule thickening, prominent trabeculae, granuloma formation surrounded by fibrous connective tissue, thickened and obstructed central arterioles, necrosis, fibrosis, and hemosiderin deposition. (Fig. 2F).

**Fig. 3:**
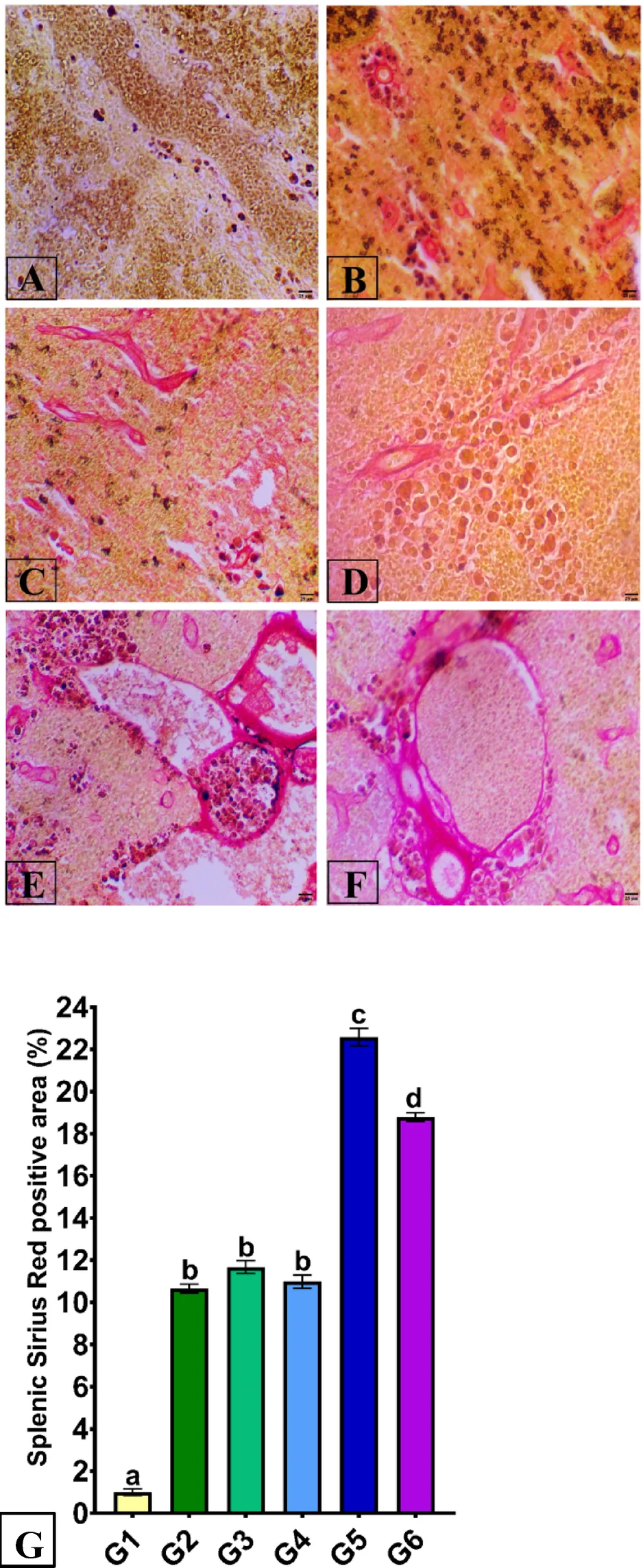
Sirius Red-stained sections of the splenic tissues of *O. niloticus* with quantitative analysis of collagen positive area (%) in the sections to depict collagen deposition in the experimental groups. **(A)** Control. **(B)** Thermal stress. **(C)** Benzene. **(D)** Toluene. **(E)** Thermal stress combined with benzene. **(F)** Thermal stress combined with toluene. Sirius Red, scale bar: 25 μm. **(G)** Collagen-positive areas were analyzed quantitatively. G1: Control (29 ± 1 °C); G2: Thermal stress (34 ± 1 °C); G3: Benzene (29 ± 1 °C + 6.1 mg L⁻¹); G4: Toluene (29 ± 1 °C + 5.9 mg L⁻¹); G5: Thermal stress + benzene (34 ± 1 °C + 6.1 mg L⁻¹); and G6: Thermal stress + toluene (34 ± 1 °C + 5.9 mg L⁻¹). Data are presented as mean ± SE (*n* = 6 fish per group). Statistical differences among groups were analyzed using one-way ANOVA followed by Tukey’s post hoc test. Bars with different letters indicate significant differences among groups at *P* < 0.05.

Sirius Red staining of splenic sections in *O. niloticus* showed minimal collagen deposition in the connective tissue of the control group. Quantitative analysis revealed significantly lower collagen-positive area in the control group compared with all experimental groups (*P* < 0.05). Thermal stress, benzene, and toluene single-exposure groups showed increased collagen-positive areas compared with the control group, with no significant differences among the individual exposure groups (*P* > 0.05). The combined exposure groups (thermal stress + benzene and thermal stress + toluene) exhibited significantly higher collagen-positive areas compared with the control and single-exposure groups (*P* < 0.05). The thermal stress + benzene group showed the highest collagen-positive area among all groups (Fig. 3).

### Reproductive hormones

**Fig. 4:**
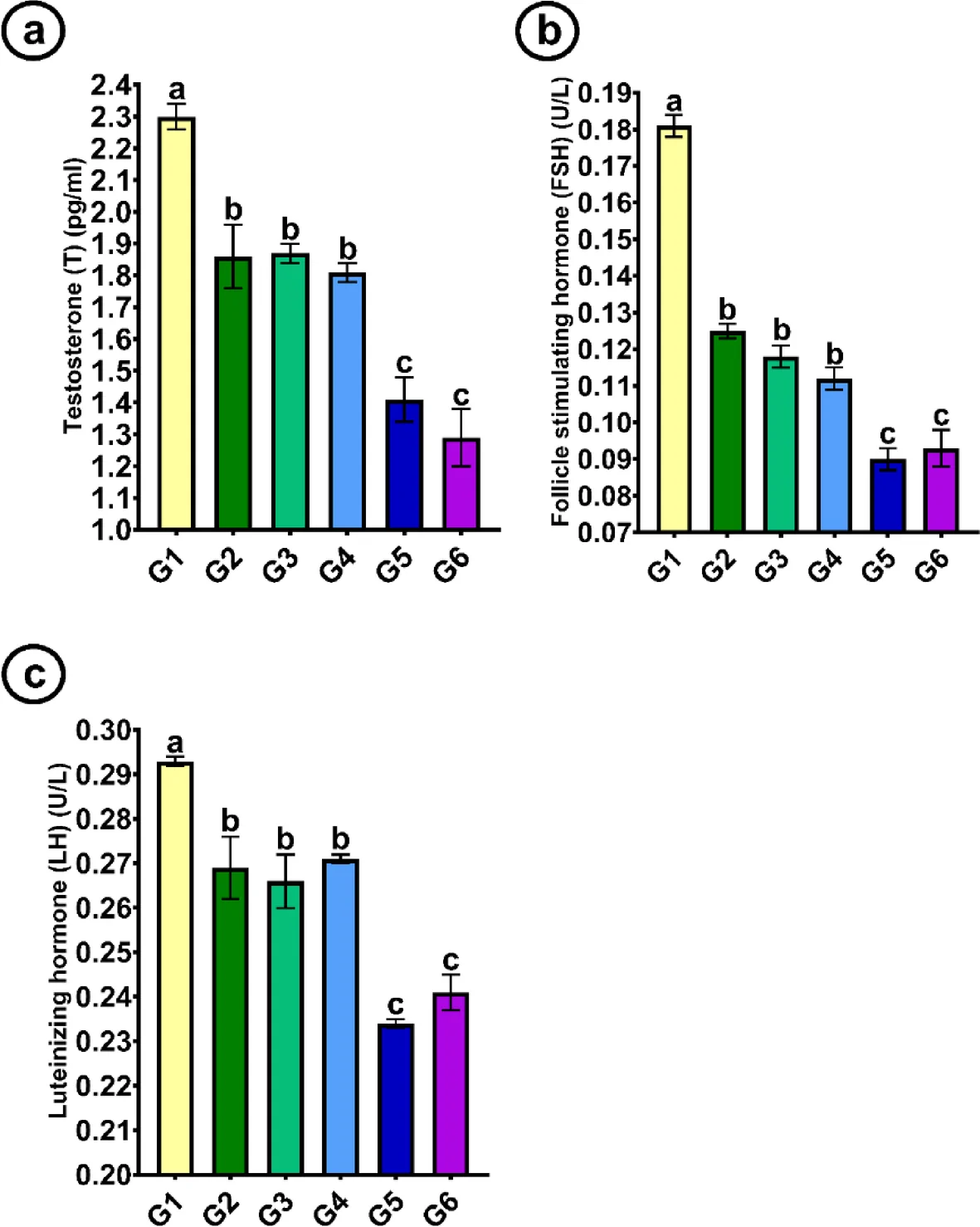
Influence of elevated temperature on the levels of FSH, LH, and T in *O. niloticus* under the influence of aromatic hydrocarbons. G1: Control (29 ± 1 °C); G2: Thermal stress (34 ± 1 °C); G3: Benzene (29 ± 1 °C + 6.1 mg L⁻¹); G4: Toluene (29 ± 1 °C + 5.9 mg L⁻¹); G5: Thermal stress + benzene (34 ± 1 °C + 6.1 mg L⁻¹); and G6: Thermal stress + toluene (34 ± 1 °C + 5.9 mg L⁻¹). Data are presented as mean ± SE (*n* = 6 fish per group). Statistical differences among groups were analyzed using one-way ANOVA followed by Tukey’s post hoc test. Bars with different letters indicate significant differences among groups at *P* < 0.05.

Reproductive hormone levels in *O. niloticus* are presented in Fig. 4. Exposure to single stressors (thermal stress, benzene, or toluene) resulted in significantly lower FSH, LH, and T levels compared with the control group (*P* < 0.05). No significant differences were observed among the single-stressor groups (*P* > 0.05). The combined exposure groups (thermal stress + benzene and thermal stress + toluene) showed significantly lower hormone levels compared with the control and respective single-stressor groups (*P* < 0.05), as shown in Fig. 4a–c.

### Histopathological and histochemical studies of testis

**Fig. 5:**
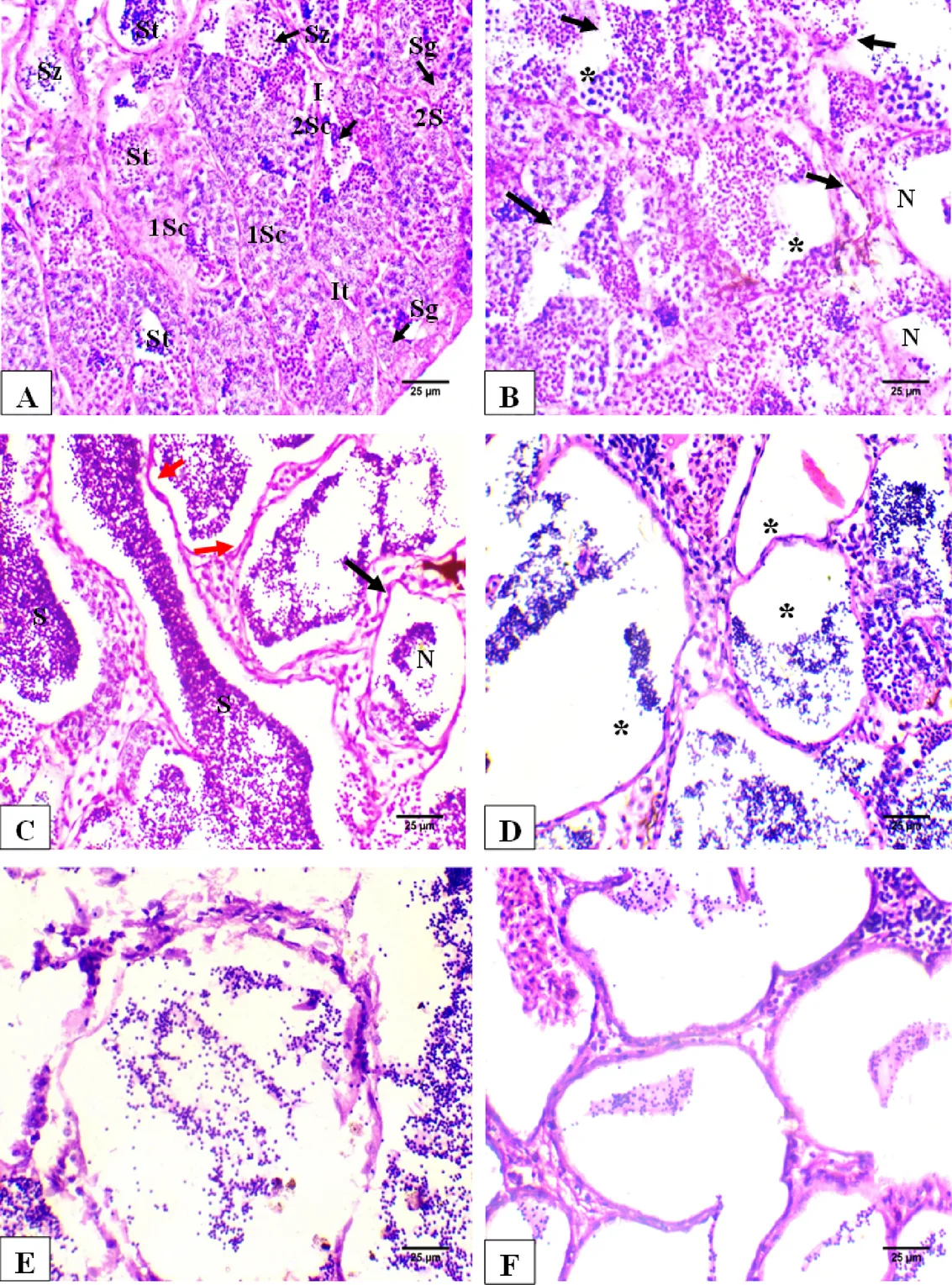
**(A)** Light micrographs of *O. niloticus* testis control sections showing normal seminiferous tubules containing spermatogonia (Sg) near the lobular wall, and cysts with primary spermatocytes (1Sc), spermatids (St), primary spermatocytes (1Sc), secondary spermatocytes (2Sc) and the interstitial tissue (It) contains capillaries. **(B)** Exposure to thermal conditions showing degenerative changes in the cellular elements of seminiferous tubules (arrows) and necrotic areas (N) with disorganization of the intratubular tissue (stars). **(C)** Testis sections exposed to benzene showing disorganization or atrophy of seminiferous tubule wall (arrow), desquamation inside the seminiferous tubule (red arrow) and distorted tubule border was observed, necrosis (N) and centralization of spermatozoa (S). **(D)** Testis sections exposed to toluene showing complete degeneration of seminiferous tubules (stars) atrophy of all sperm cells. **(E)** Testis sections exposed to thermal stress & benzene showing severe degeneration in seminiferous tubules with atrophy of all sperm cells. **(F)** Testis sections exposed to thermal stress & Toluene showing complete degeneration and severe damage of seminiferous tubules with atrophy of all sperm cells were observed. **H&E.**,** scale bar: 25 μm.**

In the current research, light micrographs of *O. niloticus* testis exhibited normal histological architecture in the control (Fig. 5A). Relative to the control group, thermal stress induced significant degenerative alterations in the cellular components of the seminiferous tubules, with necrotic foci and disorganization of the intratubular tissue (Fig. 5B). Fish exposed to benzene alone had moderate pathological changes, such as disorganization and atrophy of the seminiferous tubule wall, desquamation of the germinal epithelium into the tubular lumen, distortion of the tubule border, focal necrosis, and centralization of spermatozoa (Fig. 5C). Similarly, testis sections of fish exposed to toluene alone showed degeneration of seminiferous tubules related to atrophy of sperm cells (Fig. 5D). In contrast, fish exposed to thermal stress in the presence of benzene exhibited severe degeneration of seminiferous tubules, which is linked to complete atrophy of all sperm cells (Fig. 5E). A more severe pathological picture was detected in fish exposed to both thermal stress and toluene, where seminiferous tubules were completely degenerated and severely damaged, leading to total atrophy of all sperm cells (Fig. 5F).

**Fig. 6:**
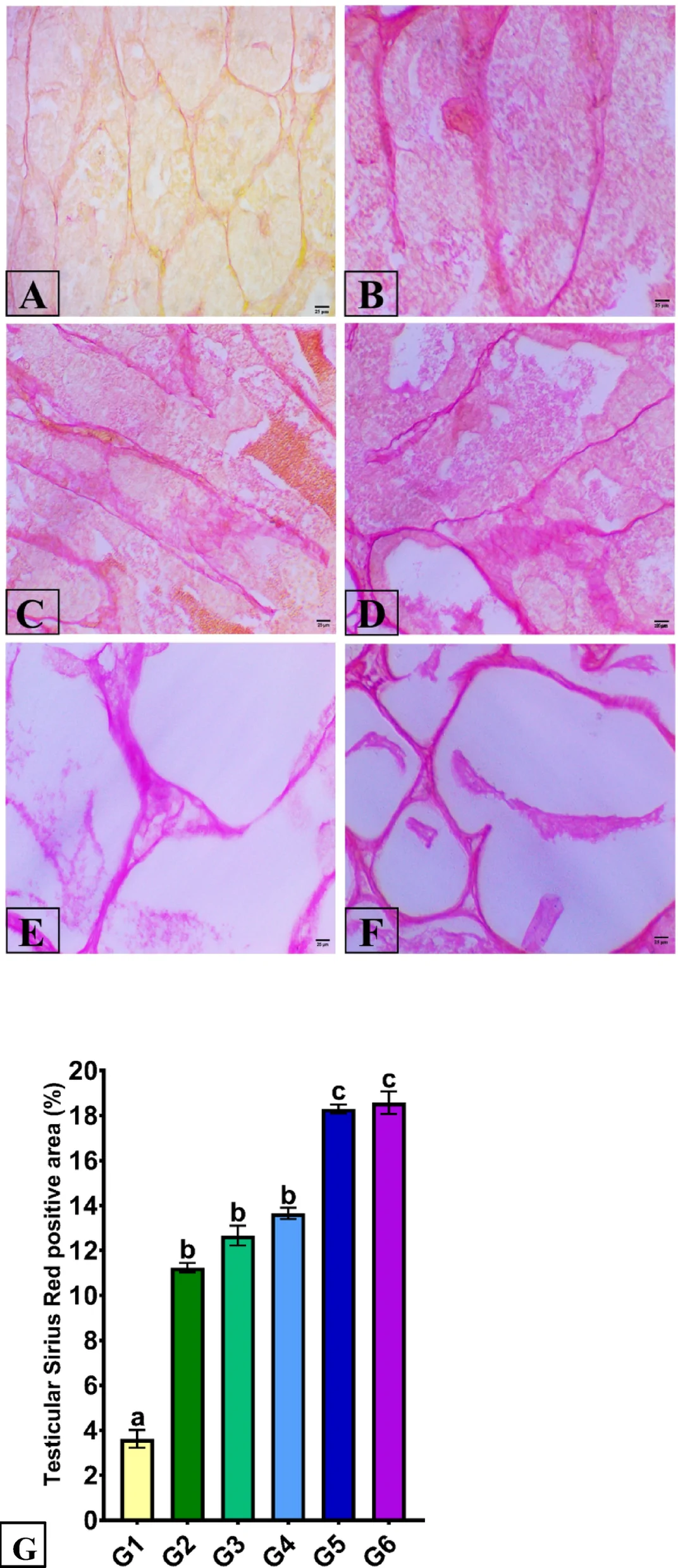
Representative Sirius Red-stained sections of testis with quantitative measurement of testicular collagen deposit positive area (%) in Nile tilapia across experimental groups. **(A)** Control. **(B)** Thermal stress. **(C)** Benzene. **(D)** Toluene. **(E)** Thermal stress combined with benzene. **(F)** Thermal stress combined with toluene. Sirius Red, scale bar: 25 μm. **(G)** Quantitative analysis of collagen-positive areas in testis sections. G1: Control (29 ± 1 °C); G2: Thermal stress (34 ± 1 °C); G3: Benzene (29 ± 1 °C + 6.1 mg L⁻¹); G4: Toluene (29 ± 1 °C + 5.9 mg L⁻¹); G5: Thermal stress + benzene (34 ± 1 °C + 6.1 mg L⁻¹); and G6: Thermal stress + toluene (34 ± 1 °C + 5.9 mg L⁻¹). Data are presented as mean ± SE (*n* = 6 fish per group). Statistical differences among groups were analyzed using one-way ANOVA followed by Tukey’s post hoc test. Bars with different letters indicate significant differences among groups at *P* < 0.05.

Sirius Red-stained testicular sections of *O. niloticus* showed minimal collagen deposition in the control group, with thin and regularly arranged collagen fibers around the peritubular basement membrane. Quantitative analysis revealed that the collagen-positive area in the control group was significantly lower than that of all experimental groups (*P* < 0.05). The thermal stress, benzene, and toluene single-exposure groups showed increased collagen-positive areas compared with the control group, with no significant differences among the individual exposure groups (*P* > 0.05). The combined exposure groups (thermal stress + benzene and thermal stress + toluene) exhibited significantly higher collagen-positive areas compared with the control and single-exposure groups (*P* < 0.05), while no significant difference was observed between the two combined-exposure groups (*P* > 0.05) (Fig. 6).

## Discussion

The overall impacts of climate-driven warming, as well as the ongoing chemical pollution, are clearly increasing in freshwater ecosystems^33^. Although there has been an increase in awareness of these stressors, their interactive impact on important biological systems, especially reproduction and immunity, is inadequately investigated. The present results indicate that combined thermal stress and benzene or toluene exposure disrupted the physiological pathways that regulate reproductive and immune competence in *O. niloticus* to a greater extent than either stressor alone. This pattern suggests that elevated temperature may increase the susceptibility of fish to benzene or toluene toxicity, resulting in broader physiological disturbances than those induced by either stressor individually. The concurrent reduction in LYZ activity and IgM, together with the elevation of IL-1β and IL-6, suggests an alteration in immune homeostasis, reflected by reduced humoral immune markers and enhanced pro-inflammatory responses following combined thermal stress and benzene or toluene exposure. Such an immune profile is consistent with patterns of contaminant-induced immunotoxicity previously described in fish^2,67^. Elevated temperature has been reported to enhance xenobiotic-metabolizing enzyme activity and increase the energetic demands associated with detoxification, potentially increasing the physiological burden of hydrocarbon exposure^68^. Under these conditions, oxidative and endocrine responses may also be relevant, as oxidative stress accompanied by increased cortisol and elevated IL-1β and IL-6 has been reported following monocyclic aromatic hydrocarbons exposure in *Clarias gariepinus*^12^. In addition, stress induced cortisol elevation has been discussed in relation to leukocyte mobilization and pro-inflammatory immune responses^69^, whereas prolonged glucocorticoid receptor signaling has been reported to suppress humoral immune effectors, including LYZ and IgM^70^. The differential response of IL-1β and IL-6 between the two combined-exposure groups may reflect differences in the biological regulation of these cytokines. IL-1β is primarily involved in the initiation of inflammatory responses, whereas IL-6 participates in a broader range of immune processes, including acute-phase responses and immune cell differentiation^71^. Differences in cytokine regulatory networks have also been described, with IL-1β reported to stimulate IL-6 production, whereas IL-6 participates in feedback regulation of inflammatory signaling^72^. This interpretation is in agreement with observations from previous studies reporting reduced LYZ activity following combined thermal stress and 4-nonylphenol exposure in juvenile *O. niloticus*^2^ and hyper salinity followed by heat stress in common carp^73^. Likewise, reduced IgM production has been reported under supra-optimal temperature conditions^74^. In addition, increased inflammatory cytokine expression, including IL-8, has been reported following petroleum-derived hydrocarbon exposure, with involvement of circulating monocytes and tissue macrophages^75,76^.

In the present study, combined thermal and benzene or toluene exposure resulted in pronounced splenic alterations, including granuloma formation, fibrosis, necrosis, vascular congestion, increased hemosiderin deposition, disruption of splenic architecture, and expansion of Melano macrophage centers. These changes were accompanied by alterations in immune parameters, suggesting an association between splenic structural impairment and immune dysregulation under combined stress conditions.

Thermal stress has been reported to affect splenic integrity in fish, with alterations including cellular degeneration, vacuolation, and architectural disruption^77,78^. Such changes have been discussed in relation to oxidative imbalance, where excessive reactive oxygen species may affect cellular membranes and mitochondrial function during thermal exposure^79^. Consistent with these observations, thermal stress has been associated with progressive splenic alterations in different fish species, including reduced cellular density, structural disorganization, and vacuolation in rainbow trout^80^, together with changes in Melano macrophage centers, hemosiderin accumulation, white and red pulp organization, and connective tissue deposition in *Clarias gariepinus*^79,81^. Similar degenerative and inflammatory features, including vacuolization and macrophage infiltration, have also been described in Siberian sturgeon exposed to chronic thermal stress^82^. Chemical exposure may further contribute to splenic alterations. monocyclic aromatic hydrocarbons exposure in *Clarias gariepinus* was associated with changes in lymphoid follicles and persistent splenic alterations following exposure, which indicates the long-term effect of the reactive oxygen species -mediated cellular damage and inflammation^12^. In addition, benzene exposure has been linked to splenic pathological changes, including alterations in hematopoietic tissue, lymphoid organization, necrosis, and cellular degeneration^83–85^. Together, these observations provide a biological context for the splenic lesions observed in the present study and suggest that simultaneous thermal and benzene or toluene exposure may impose additional structural and immunological challenges to the spleen.

The concurrent immune alterations observed in the present study were accompanied by marked disturbances in reproductive endocrine function, reflected by reduced FSH, LH, and T concentrations, together with pronounced testicular lesions. These findings suggest that combined thermal stress and benzene or toluene exposure affected both immune and reproductive regulation to a greater extent than either stressor alone.

Elevated temperature has been reported to interfere with the hypothalamic–pituitary–gonadal axis, reducing gonadotropin secretion and steroidogenesis, thereby impairing male reproductive function through endocrine disruption^86–89^. In parallel, thermal stress has been linked to oxidative stress characterized by excessive reactive oxygen species production and mitochondrial dysfunction, which may impair cellular energy metabolism and gamete quality despite the induction of heat-shock proteins^90^.

Chemical exposure may further impair reproductive function through endocrine disruption and oxidative tissue injury. Benzene has been associated with seminiferous tubule degeneration, reduced T production, oxidative stress, mitochondrial dysfunction, inflammatory activation, and altered RNA methylation that may interfere with germ-cell differentiation and spermatogenesis^91,92^. Similarly, toluene exposure has been associated with impaired early spermatogenesis, oxidative damage, DNA injury, reduced sperm quality, and decreased T concentrations^93,94^.

The reduction in FSH, LH, and T concentrations was accompanied by pronounced testicular histopathological alterations, including degeneration of seminiferous tubules, necrosis, disorganization of intratubular architecture, germ-cell desquamation, and marked depletion of spermatogenic cells. These lesions were more severe under combined thermal and benzene or toluene exposure than under the corresponding single-stressor conditions.

Similar testicular alterations have been reported following thermal exposure, including seminiferous tubular atrophy, loss of spermatogenic cells, degeneration of Sertoli and Leydig cells, and disruption of spermatogenic organization in *O. niloticus*^95,96^. Aromatic hydrocarbons may further contribute to these alterations through oxidative tissue injury and impaired spermatogenesis. Consistent with this interpretation, comparable lesions have been described following benzene, toluene, ethylbenzene and xylene exposure^97^, benzene exposure^92^ and toluene exposure^94^, supporting the patterns observed in the present study.

Sirius Red staining demonstrated increased collagen deposition in both splenic and testicular tissues following combined thermal and benzene or toluene exposure. This fibrotic response may reflect oxidative tissue injury, where excessive reactive oxygen species production and inflammatory activation contribute to extracellular matrix remodeling and collagen accumulation. Similar mechanisms have been reported following petroleum hydrocarbon exposure^98,99^ and under thermal stress, where oxidative tissue injury has been associated with structural degeneration and collagen deposition in splenic and testicular tissues^79,81,100^.

## Conclusion

Collectively, the present findings indicate that combined exposure to elevated temperature and benzene or toluene was associated with more pronounced immune, reproductive, and histopathological alterations in *O. niloticus* compared with exposure to either stressor alone under the experimental conditions of this study. These findings suggest that elevated temperature may modify the physiological responses of fish to benzene or toluene exposure, emphasizing the importance of considering multiple environmental stressors in aquatic toxicological assessments.

Several limitations should be acknowledged. The study was conducted over a relatively short exposure period and under controlled laboratory conditions, which may not fully represent complex environmental scenarios. Furthermore, the investigation was limited to a single fish species, and histopathological evaluation was based on qualitative observations without a semi-quantitative scoring system. In addition, only one exposure concentration was used for each hydrocarbon, which limits the assessment of concentration–response relationships. Future studies incorporating longer exposure periods, multiple exposure concentrations, additional species, environmentally relevant conditions, and semi-quantitative histopathological assessments are recommended to further clarify the combined effects of thermal stress and benzene or toluene.

## Data Availability

All pertinent raw data will be made freely available by the corresponding author upon request.
